# Supplementary material for: Patterns of Intron Gain and Loss in Fungi
Source: PLoS Biol. 2004 Nov 30;2(12):e422. doi: 10.1371/journal.pbio.0020422 (PMC532390; doi:10.1371/journal.pbio.0020422)
Supplement: Table S1 — Also available at http://genes.mit.edu/NielsenEtAl/. (4.3 MB ZIP). [file pbio.0020422.st001.zip › NielsenEtAl/html/523.html]

AN4706.1.NCU00551.1.MG03060.1.FG08719.1


```
 CLUSTAL W (1.82) Multiple Sequence Alignments - Introns Inserted


Sequence 1: NCU00551.1	2397 aa
Sequence 2: MG03060.1	2403 aa
Sequence 3: FG08719.1	2398 aa
Sequence 4: AN4706.1	2404 aa
Alignment Length: 2447 aa
Number Identitical Residues: 1244 aa
Alignment Score (without introns) 58986


MG03060.1 	------MSGSLRNNPFMRGASPSQSSSSPAGTPKSRPKSTAFP-PASLPFSNASTATGAL
NCU00551.1	--------MSVRNNPFARNNAPPQQGPNAPG--LGRPRSAIFTNPAAPVMPSAGPAAGAT
FG08719.1 	MSFNANGTAQRRSNPFARTASASPATSTNPR-----PKSAIFTSPSSTPGIVSSFGHNRT
AN4706.1  	MLPSQINGSPKRANPFSRASPSPSPTPSPQT--KARPKSAVLASAGKFEEPKSHIRN---
          	   .       * *** *  ...   ..      . *:*: :....      :       

MG03060.1 	AHP----TRTQSHTTN---VLIPRHTREGSRGAAS-SSTFAPSFIKSEEMRR-SDTVRGI
NCU00551.1	PPTPPTHIRSLSHASSTPTLVQPIPIRPGQKSSLPGSTTFAPSFIKTDDMRKHADIVKGI
FG08719.1 	QSNGS--VSAISREFS----SAQTRLRSDSKSGTPTSTTFAPSFIKEEEMIRSQDEVQGI
AN4706.1  	-------SSSISHTSLAPFGRNANRQRSNSLRNDVTSGTFAPEFIKSEDLRHGADQIRGQ
          	         : *:   :.        * ..      * ****.*** ::: :  * ::* 

MG03060.1 	EGENDFSGKRYVWLKDPQTAFVKGWVVEELGAGRILVQCDDGT0QREVDSESVDKVNPAK
NCU00551.1	EGE-DFSGKKYVWLKDPQAAFVKGWVVEELPENKLLVQCDDGS0QREVDAETVDKVNPAK
FG08719.1 	EGENDFSGKRYVWLKDPQTAFVRGWIVEEKDGNQLLVQCDDGT0QREVDAESVDKVNPAK
AN4706.1  	EGDNDFSGNKYVWLRDPEKAFVKGLVLEEQDGARLLVQTDDGQ0QREVDVDQVDRVNPAK
          	**:.****::****:**: ***:* ::**    ::*** ***  ***** : **:*****

MG03060.1 	FDKAEDMAELTHLNEASVVHNLHMRYQSDLIY~TYSGLFLVTVNPYCPLPIYSNEYVNMY
NCU00551.1	FDKANDMAELTHLNEASVVHNLHTRYQSDLIY~TYSGLFLVAVNPYCPLPIYTNEYINMY
FG08719.1 	FDKANDMAELTHLNEASVVHNLHMRYQTDLIY~TYSGLFLVTVNPYATIPIYNNDYINMY
AN4706.1  	FDKADDMAELTHLNEASVVHNLHTRYLADLIY0TYSGLFLVTVNPYCPLPIYSNEYINMY
          	****:****************** ** :**** ********:****..:***.*:*:***

MG03060.1 	KGRSREDTKPHIFAMADHAFRNLVDEGENQSILVT~GESGAGKTENTKKVIQYLAAVAHS
NCU00551.1	KGRNREDNKPHIFAMADEAFRNLVDEHENQSILVT~GESGAGKTENTKKVIQYLAAVAHP
FG08719.1 	RGRSREDTKPHIFAMADEAFRNLVEEGKNQSILVT~GESGAGKTENTKKVIQYLAAVAQA
AN4706.1  	KGQSREETRPHIFAMADEAFRNLVEEGENQSILVT2GESGAGKTENTKKVIQYLAAVATS
          	:*:.**:.:********.******:* :******* ********************** .

MG03060.1 	ESPVKAKGQSQHSNLSAQILRANPILEAFGNAQTVRNNNSSRFGKFIRIEFSKNGSIAGA
NCU00551.1	DPSGRTRG-SQHSNLSAQILRANPILEAFGNAQTVRNNNSSRFGKFIRIEFSSNGSIAGA
FG08719.1 	DSTIKNKA--QHSSLSEQILRANPILEAFGNAQTVRNNNSSRFGKFIRIEFTRNGAICGA
AN4706.1  	DNMYSRSGSKQMNTLSQQILRANPILEAFGNSQTVRNNNSSRFGKFIRIEFSRSGQISGA
          	:      ...* ..** **************:*******************: .* *.**

MG03060.1 	FIDWYLLEKSRVVRINSNERNYHVFYQLLKGADRRMKEDFLIDGMDTEDFAYTRDGNDTI
NCU00551.1	FIDWYLLEKSRVVHVNANERNYHIFYQLLKGADRKLKQELLLDSMDVKDFTYTRDGHDTI
FG08719.1 	YIDWYLLEKSRVVHINQHERNYHIFYQLLTGASSSLKREFLLNDLTVKDFVYTRDGHDSI
AN4706.1  	SIDWYLLEKSRVVKPNLQERNYHIFYQLLRGAEPKLKQKLLLSNLQIEDFAYTREGNDTI
          	 ************: * :*****:***** **.  :*..:*:..:  :**.***:*:*:*

MG03060.1 	SGVSDREEWETLMEAFHVMGFNGQDQAAILRTIAAVLHLGNINLVKESRAADQARLAPDA
NCU00551.1	TGVSDKEEWDSLIEAFLVMGFSDEEQVAILRTIAAVLHLGNITVVKESRSADQARLNPDA
FG08719.1 	VGVSDPREWASLMEAFNVMGFSDDEQKSILRTVAAVLHLGNINVVKESRTADQARLAPDA
AN4706.1  	AGVSDEKEWDSLLEAFHIMNFSEEDQMCILRTVAAVLHLGNITIVKESLRADQAALSRDA
          	 **** .** :*:*** :*.*. ::* .****:*********.:****  **** *  **

MG03060.1 	KSFAEKVCRLLGIPLEPFLQGLLHPKVKAGREWVEKVQTPEQVRLGIDALSKGIYERAFG
NCU00551.1	KAIAGRACKLLGVPLEPFLQGLLHPRVKAGREWVEKVQTPEQVRLAIDALAKGIYERGFG
FG08719.1 	KAQAAKVCKLLGVPLEPFMRGLLHPKVKAGREWVEKVQTPEQVRLSLDALSKGIYERGFG
AN4706.1  	LDSVHKACQLLGIETEPFVKGLLHPKVKAGREWVEKVQTPEQVRLALDALAKGIYERGFG
          	   . :.*:***:  ***::*****:*******************.:***:******.**

MG03060.1 	DLVTRINRQLDRTGMGMDDSRFIGVLDIAGFEIFEENSFEQLCINYTNEKLQQFFNHHMF
NCU00551.1	DLVARINRQLDRTSMGLDDTRFIGVLDIAGFEIFETNSFEQLCINYTNEKLQQFFNHHMF
FG08719.1 	DLVTRINRQLDRTGMGLDDSHFIGVLDIAGFEIFEQNSFEQLCINYTNEKLQQFFNHHMF
AN4706.1  	DLVNRINSRLERNTVTGEDSYFIGVLDIAGFEIFQNNSFEQLCINYTNEKLQQFFNHHMF
          	*** *** :*:*. :  :*: *************: ************************

MG03060.1 	VLEQEEYAREQIEWQFIDFGRDLQPTIDLIELPNPIGIFSCLDEDSVMPKATDITFTEKL
NCU00551.1	VLEQEEYAREQIEWKFIDFGRDLQPTIDLIELPNPIGIFSCLDEDCVMPKATDKTFTEKL
FG08719.1 	VLEQEEYAREQIEWKFIDFGRDLQPTIDLIELSNPIGVFSCLDEDCVMPKATDKSFTEKL
AN4706.1  	VLEQEEYAREQIEWQFIDFGKDLQPTIDLIEVTNPIGIFSCLDEDCVMPKATDKSFTEKL
          	**************:*****:**********:.****:*******.******* :*****

MG03060.1 	HSLWDKKSNKYRPSRLKHGFILTHYAAEVEYSTDGWLEKNKDPLNDNVTRLLAASTENHV
NCU00551.1	NSLWDKKSQKYRPSRLGQGFILTHYAAEVEYSTEGWLEKNKDPLNDNITRLLAASTDKYM
FG08719.1 	NSLWDKKSTKYRPSRLGQGFILTHYAAEVEYSTEGWLEKNKDPLNDNITRLLAASTNEHV
AN4706.1  	HSLWDTKSTKYRASRLRQGFILTHYAAEVEYSTDGWLEKNKDPLNDNITRLLASSQDNHI
          	:****.** ***.*** :***************:*************:*****:* ::::

MG03060.1 	ANLFADCAEQDDEYGGVRSRVKKGLFRTVAQRHKEQLSSLMAQLHSTHPHFVRCILPNHK
NCU00551.1	ANLFSDCADQDDEAGAMRSRVKKGLFRTVAQRHKEQLSSLMTQLYSTHPHFVRCIIPNHK
FG08719.1 	ASLFSDCAESEEDRATGRSRVKRGLFRTVAQKHKEHLHSLMSQLHLTHPHFVRCILPNHK
AN4706.1  	AALFSDCG-NADEVDHPRSRVKKGLFRTVAQRHKEQLSSLMNQLHSTHPHFVRCIIPNHK
          	* **:**. . ::    *****:********:***:* *** **: *********:****

MG03060.1 	KKPKMFNNLLVLDQLRCNGVLEGIRIARTGFPNRLSFSEFRQRYEVLCRDMPKGYLEGQA
NCU00551.1	KKPKQFQSLLVLDQLRCNGVLEGIRIARTGFPNRLPFAEFRQRYEVLCPNMPKGYVEGQA
FG08719.1 	KKPKEFNNLLVLDQLRCNGVLEGIRIARTGFPNRLPFAEFRQRYEVLCQNMPKGYLEGQA
AN4706.1  	KRPKMLNAPLVLDQLRCNGVLEGIRIARTGFPNRLSFNEFRQRYEVLCRDMPKSYMDGQS
          	*:** ::  **************************.* ********** :***.*::**:

MG03060.1 	AASIILDKLGLDRGLFRVGLTKVFFRAGVLAELEEQRDALITEIMSRFQSVARGFMQRRI
NCU00551.1	AARIMLDRFGLDKSLYRVGLTKVFFRAGVLAELEEKRDAIITEIMAKFQSLARGYIKRRI
FG08719.1 	AASLMLEKLGLDRSLYRVGLTKVFFRAGVLAELEEQRDALITQIMSRFQSVARGFVQRRA
AN4706.1  	AARIMLQKLALDKAWFRVGRTKVFFRAGVLAELEEKRDELIRTIMTRFQSVARGFVQRRI
          	** ::*:::.**:. :*** ***************:** :*  **::***:***:::** 

MG03060.1 	AYKRLYRAEATRVIQRNLNVYLNLCDDPWWQLLIRMKPLLGATRTATEVKRRDELIRQLH
NCU00551.1	AYKRLYRAEATRVIQRNFQVYLELSENPWWQLMVRMKPLLGATRTATEVKKRDEMIRQLS
FG08719.1 	AYKRLFRTEATRIIQRNFNVYLDLAENPWWQLIVKMKPLLGTTRTATEVKRRDAMIKELN
AN4706.1  	SNKRLYRAEATHIIQHNFRAYLEMKANPWWRLFSRMKPLLGETRTAQEVKRRDEKIKQLE
          	: ***:*:***::**:*:..**::  :***:*: :****** **** ***:**  *::* 

MG03060.1 	DKMKQEEMDRQRLEEERRTTHTEMMRIQQTLESERALALDKEEIFKRLQLREVELEEKLS
NCU00551.1	DRMKQEAMDRQRLEEERRNVHAEMLRIQQTLESERALALDKEEIFKRLQLREAELEDKLA
FG08719.1 	DKMQVESENRLKLEEERRNCHAEMVRIQQTLESERALALDKEEIFKRLQLREAELEEKLA
AN4706.1  	TKMKQDQSERQKVEEERRRAEIEIQRIQQTLESERALALDKEEIFKRLQDREVELSEKLA
          	 :*: :  :* ::*****  . *: ************************ **.**.:**:

MG03060.1 	GAIEDQERLEDQLDSLLDAKKLAEEQVEKYRTQLEQAASLIGKLEEEKTDLGDRLSSLEK
NCU00551.1	GALDDQERMEEQMDGLLEAKKRAEVDVEKYRTQLEQAATIIGRLEDEKSELANRIADLER
FG08719.1 	GAIEDQERLEDELDDLLNAKNRAERDVETFRSQLEQAATLIAKLEEEKNGLSTKIIELEK
AN4706.1  	GAIADQENLEDQLDELILAKKKTDEELDLRKTQLEQAGEIIQRLEAERKEMQQKLEDLEQ
          	**: ***.:*:::* *: **: :: :::  ::*****. :* :** *:. :  :: .**:

MG03060.1 	EMKEMSQRQSQRSAEEEALQDEIKMLQSQLSLKDRKARDLEGKLLKIDQDAEVKLVAAQK
NCU00551.1	QIVEISQKQSERSEQETALENELKMLQSQLSLKDRKVKDLESKLLKIDQDSDVKLLATEK
FG08719.1 	SITEMTQKQAERSEQEVLLQDEIKMLQSQLAVKERKAQDLENKLVKLDQDLELKLHIAQR
AN4706.1  	KLLEAQSSASETENHMRELGQEVKMLQSHLSLKERKLQDLEAKLLKTDQDLDVKLAKTSK
          	.: *  .  :: . .   * :*:*****:*::*:** :*** **:* *** ::**  :.:

MG03060.1 	ELQTFKSREQRLIAENRDAQQQLSKLSKTSTDYEDLVRKKESELALLRSDNKKYETEHRS
NCU00551.1	ELQIAKSKNEQLTTENTQFQQQLTQLSKTSTSYEDLIRKKESELAILRSENKQLATERRS
FG08719.1 	DLQAMKLRETQLSQENDDIQQQLNELSKTATEYEDLVRQKESELSLLRTDNKNFESERQS
AN4706.1  	ELDRSKKEVKDLLDENRTIRQQISDLSKTSTSYEEMLRRKESELTVLRNDAKKHAEEKKQ
          	:*:  * .   *  **   :**:..****:*.**:::*:*****::**.: *:   *::.

MG03060.1 	FEEQKKTLAAEKEKAATRLREVQAEISALKSQQSQLQREADDAKKLLQARLSEDAQADQN
NCU00551.1	IDDQTKMLASEREKAAERLREVQAELTAMKSQQAQLMREAEDAKKLLQARLSEDAQADQN
FG08719.1 	LEDQRKSLTAEKEKNAEKYHEAQAELVAMKSRQAQLEREAEDAKNLLEARLSEDAQADQN
AN4706.1  	LDVEKSNLSTRHDNMQNKLRELQAQVDAMVSEKIQLEREAADVQKLLQAKISEDAEAGES
          	:: : . *::.:::   : :* **:: *: *.: ** *** *.::**:*::****:*.:.

MG03060.1 	RQVLEGQIKDLKDELYKTQMDLSRERQSRDDVLLLGEHEYQKLQEEYERLNESKITIEKE
NCU00551.1	RQVLEAQIKDLKDELYNAQMELSRERQSRDDVQLLGEHKYQTLKDEYNHLNESKITIEKE
FG08719.1 	RQVLESQTKDLKDELYKTQMELSRERQSRDDVQLLGEHRYQELKDEFDRVNESKIIIEKE
AN4706.1  	RKLLEQQIQDLKNQLFQAQADLSRERQSRDDVQMLAEHKLAELKDKFESLNEAKITIEKE
          	*::** * :***::*:::* :*********** :*.**.   *::::: :**:** ****

MG03060.1 	LYAQQDTLRRTMEARATAEKERDEARDEIRKLRVAKVQAEEARREAENAGERAALKIARE
NCU00551.1	LYAQQDTLRRAVETRTTLEQERDDAREEIRRLRSAMTQAEEARREAEVLGERAATKIARE
FG08719.1 	MYAQQDTLRRTLEARTTAEKERDEARQEIRRLRVAKTQAEEARMQAEVAGERQASKAAQD
AN4706.1  	MYIQQDSLRRATEARVAAEQSRKELQQELIKLRDRFTAAEDARLRAEAEMEQALVKQTTQ
          	:* ***:***: *:*.: *:.*.: ::*: :**   . **:** .**   *:   * : :

MG03060.1 	RETSLRKDLDAAQERLKWFEDECAKLNHQVEDLNKLILSSGEFGLKNDQEKERMQRELTT
NCU00551.1	REESLLKDLDAANDKLKWYESECAKLNYQVEDLNKVIIESGEFGLKNDQAKERLERELNT
FG08719.1 	RENSLRKDLDAAQERLHWFDGECAKLNHQIEDLNKLILESGEFGLKNDQAKERLERELTT
AN4706.1  	RLESIRKELDNTQHRLEEVEAERSRLAAREQQLMNAIAESESFRIRHDQHKERLERELVT
          	*  *: *:** ::.:*.  : * ::*  : ::* : * .* .* :::** ***::*** *

MG03060.1 	VKGRLMASENDNRALLNKLQQKGLEIARSTSRASEASRGQVTALSREKARVEEQNVKLNK
NCU00551.1	VRSRLAASENDNRALLNKLQQKGLEIARSASRAGEASRAQLVQVQREKTRVEEQNTKLNK
FG08719.1 	VKSRLTASENDNRALLNKLQQKGLEIARSSSRASEASRGQIQSLQREKARLEEQNTKLNK
AN4706.1  	LKGRLTASENDNRALLTKIQQKNLDIARSNSKASESQRLRVATLQKEKAKLEEENKALSR
          	::.** **********.*:***.*:**** *:*.*:.* ::  :.:**:::**:*  *.:

MG03060.1 	QLGDAQLTIAALEKKAEKLQLNLEDLNLEVSRETKASRNAEKTSSNFTAQLAEANRTIES
NCU00551.1	QLGDSQVTVAGLEKKVEKLQLTIEDLNHELARETQASRNAEKLSSNVTVQLAEANRTIES
FG08719.1 	QLGDSQLAIASLEKRAEKLQLNLEDLSHEVAREVKSSRNAEKATSTFTAQLAEANRTIES
AN4706.1  	QLGDQQLSITSLEKQKEKLALSVEDLNHEVNREHKASRNAERAASTASIQLAEANRNLET
          	**** *::::.***: *** *.:***. *: ** ::*****: :*. : *******.:*:

MG03060.1 	ERQLKTQAQTTVRTLQTTLDSRDKELVELRAQMLSILKTVDPDALPAIAPDN--TKNEMF
NCU00551.1	ERQLRTQAQGTVRTLQSTLDARDKELIELRAQLLSALKTVDPDTDDTHVQTEGASASERF
FG08719.1 	ERQLRTQAQTTVRTLQSSVDSRDKEINELRAQMLEVLRTVDPEVHIPPQSDG---TDEKV
AN4706.1  	ERQLRTQAQANTRKLQSALDSSNKEIDDLHRQLMLFHKIVEPEADPSADSWD---KIQPD
          	****:**** ..*.**:::*: :**: :*: *::   : *:*:.  .          :  

MG03060.1 	IAKNYDLARKVEELQQSLRVQTAARQTAENQVAELR----------AQKHDSP-RPRLEE
NCU00551.1	LSKNFDLVRKIEDLQQNLRVQTTARASAEAQLADLR----------TSRNDSPTRPRLEE
FG08719.1 	LIQNFDLVRKVEELQQNLRVQTAARTNAENQLTEMR----------ASRNESPTRPKLEE
AN4706.1  	LSRKVDLAQLVETLQSQLQVSEEKYNRAEAQLAEMRRRHGEEMKELDARYSSSKRALLEE
          	: :: **.: :* **..*:*.      ** *::::*   ... ..   : .*..*. ***

MG03060.1 	INPNEAPFNVSPTQRKS---KINGRHYSTASTPPR--RFNGADGDHSHDSVRSDRTADVL
NCU00551.1	ISPNEAPFTGSPTQKR----KTNGRHHSNASTSPRERRFTNSDSENLHDSVRSDRTADIV
FG08719.1 	IQLNEAPFQGSPTQRRTT--KLHALNHSNTSTPTR--RFQEPD--HLQDSTRSDRTIDTL
AN4706.1  	IDQNEVAHNRTPNHLRKNSETAIAKKFGAPTTPNR--RLNIND--NSNDSARSDRTVDTV
          	*. **...  :*.: :..:..  . :.. .:*. *  *:   *  : :**.***** * :

MG03060.1 	SFNNRQDLKTEVEELQNQLQLSEMKNRHLESQLERATPGPDATYGEESPSARRRAQKLEQ
NCU00551.1	SFNTRMDFKAEIEELQNQLQLAQMQNRHLQSQLERTASGEEDAYLDESPQLRR-VQKLEK
FG08719.1 	NFNNRMDLKAEVEELQNQLQLKEMQNRHLQSQIDRGTPVPEN--HDESPSLRR-IQKLEM
AN4706.1  	GYQKRMDLAAEIEELQNKLQMSEMQNKHLQDQLARSVP-PKDIWQDDSPSIRR-MQLLER
          	.::.* *: :*:*****:**: :*:*:**:.*: * ..  .    ::**. **  * ** 

MG03060.1 	ANSRLQDMLDDSSKKVSALEKTVRTGELSLRDIQTRSHEEILDLLNSQEESRRSLLLSHR
NCU00551.1	ANNRLHELLDDSSKKVSALEKALRTGELSLRDIQTRSHEEILDLLNSQEDSRRSLLHSHK
FG08719.1 	VNTRLHEMLDDSNKKVSALERTIRSGQLSLRDVQTRTHEEILEVLNSQEDSRRALVHSHK
AN4706.1  	ENGRLHEQLDDSSKKVSALERSIHSGELSLRDVQAKSHEELYDLINSQEQSRRSLLRVHN
          	 * **:: ****.*******:::::*:*****:*:::***: :::****:***:*:  *.

MG03060.1 	DAVAELTDIKEHFDKMRHDRAKVEVDLRDARSELQDISLARDQEAASRSQLLQEFADLQI
NCU00551.1	DAVAELTELKEHYDKVRHERAKLEVDLRDARSDLQEMSLARDQEAASRSQLLQEFADLQI
FG08719.1 	DAVAELTDIKGHFERLRHDRAKAEVELRDVKSDLEEMTLAREQEAQSRNQLLQEFADLQI
AN4706.1  	ETVAEITDAKAQFEKLKRAKAALEVELRDACSEAQELQAAREQDALSRNQLLQEFSDLQI
          	::***:*: * ::::::: :*  **:***. *: :::  **:*:* **.******:****

MG03060.1 	RLDAETSKLAD~VTANLHLYKSRADEYFSKLEQAEIAVLKANRAEQFARSQAREAEDTCA
NCU00551.1	RLDAEASKLAD0---------------------AEIAVLKANRAEAFARAQAKEAEDSCA
FG08719.1 	RLDAETSKFID~VASSLEMYRSRADEYFSKLEQAEIAVLKASRAEQFARSQAREAEDTYV
AN4706.1  	RLDAETSKSAD~LAASLSLYKSRADEYFSKLEQAEITLLKASRAEQFAKAQAKETEDNCA
          	*****:**  *  ::.     : :..  :. ..***::***.*** **::**:*:**. .

MG03060.1 	EVMSERQRMDAAVEDLQRQNQRLEEKLEDLSTDLEAATQAKRRLQHELEDYRAQRANDIE
NCU00551.1	EMMSERKRLDTMIEDLQHSNQRLEEKIEDMSTDLAAVTQAKKRLQHELEDYRNQRANDIE
FG08719.1 	EIMAEREKMDATIEDLQRQNQHLEEKVEDISTDLAAATQAKKRLQHELEDYRNQRAIDIE
AN4706.1  	QIMAERKEMEAIIDDLQRQTQSLEARMEDQAAELQGALQAKQRLQNELEDYRNQRAIDIE
          	::*:**:.::: ::***:..* ** ::** :::* .. ***:***:****** *** ***

MG03060.1 	DKESSMEQTRKKYQAEFATLTKELDLAREERLFKQAEVVRLREELDELRSKWDDEVLNSS
NCU00551.1	DKESSMEQMRKKYQAEFATLANELDRAREEKLRKQSEITRLREELDELRSKWDDEVLNSS
FG08719.1 	DKESSMEQTRKKYQAEFATLTNELDLAREEKLFKQAEIARLREELDELRSKWDDEVLNSS
AN4706.1  	DKETSMEQTRQKYQREITTLNNELEMEREKVLNGRTEASRLREELEDLRSKWDNEVLNSS
          	***:**** *:*** *::** :**:  **: *  ::*  ******::******:******

MG03060.1 	TWSKEKTRLEATLADVVASRDEAVGAHNEAQGKIVNLLSQVRTLRANVDEASAERDALLR
NCU00551.1	TWSKEKARLEATLSDVVASRDEAVSAHNEAQSKIVTLLGQVRSLRSSLDEITAERDALFR
FG08719.1 	TWCKEKARLESTLADVASSRDEAVNAHNEAQGKVVTLLSQVRSLRSSVDEIAAERDHLIR
AN4706.1  	TWAKEKSRMEVMLQDVTTSRDEAVNAHNDAQARVVSLLSQVRSLRTSIDDVTAERDMLHK
          	**.***:*:*  * **.:******.***:**.::*.**.***:**:.:*: :**** * :

MG03060.1 	EKRGLESRLQEAKAGLEELSKAGESPSLRNAAGMDKEILELKSSLAHHEDVASAAVEKMR
NCU00551.1	EKRSIEARLEEAKAGLDELANS-ESPSLRNAAHMDREILELKSSLAQQEDIAAAAVEKMR
FG08719.1 	EKRNVEARLEEAKSGLEDLVKG-ESPSLRNAANMDKEILDLKSNLAQQEDIAAAAVEKMR
AN4706.1  	EKKMLEARLTEAGERLEDLAKG-ESPSMRNAASMDRELLELKAKLAQQEDLSLAAVGKMR
          	**: :*:** **   *::* :. ****:**** **:*:*:**:.**::**:: *** ***

MG03060.1 	RAEALVAEVQKDMAAERDNAAKLQKEKTALEKALSEEKVKLVNLETKVYPSAG-QVKFLN
NCU00551.1	RAEALAAEVQKDIAAERENNADLQKQKVTLEKNFNELQMKLIDLETKGYSTASHDIKFLH
FG08719.1 	RAESLVAEVQKDVMVERENSIKLQQQKASLEKVLNEAQLKLVDLETKGYSSASQDVKFLH
AN4706.1  	RAEALATEMQKEVTAEREATAQLFKDKAALEKQLKEAQLRCVDLETKSYSSGSQDIRFLH
          	***:*.:*:**:: .**:   .* ::*.:*** :.* ::: ::**** *.:.. :::**:

MG03060.1 	TRIEE0AQ---EEQHN---KSQRSVRNVDRTVKDLQSQIERKDKQNGQLQDDLTRMRDKA
NCU00551.1	SRIKE0LESQLEEQENERSKSQRSVRNVDRIVKDLQSQIDRKDKQNTQLQEDVTRMRDKV
FG08719.1 	KRIQE0LESQLEDQETERSKSQRSVRNVDRIVKDMQGQIDRKDKQNMQLSDDVSRMRDKV
AN4706.1  	KRIKE0LETHLEEQEAKNNSEQRSLRNVDRTVKDLQSQIERREKMNAQLEEEVNRGREKV
          	.**:*  ::  *:*. . ...***:***** ***:*.**:*::* * **.:::.* *:*.

MG03060.1 	DKLLKTIDELQAADSTAQLNARRAERELREEKEKTMQLERELESWKASAPRPGSSSISG-
NCU00551.1	DKLLKTIDELQSSESANELSARRAERELREEKEKALRLERELEGWKNLRMEKGSAMGGGG
FG08719.1 	EKLLKTIEELQSSESNNQLSARRAERELREEKERAIRMERELAGLKSLRNDKGS------
AN4706.1  	ERLLKNIDELQQSESDSQLQARRAERELREEREKSLRLERELNGWKALRVERGG------
          	::***.*:*** ::*  :*.***********:*:::::**** . *      *.      

MG03060.1 	--YGSLRRGPNGTINSRSGFGSLEGVDEFGGVEIPKRKSSISRAPSLTKGFL
NCU00551.1	GGSGSVAGSVLGSIRGKRTFTN-DNMQMDGVIEIPKRKSSISRAPSLTKGFI
FG08719.1 	-------GSVMGSVRSRMGPWRVSEGDDASMIDVPGRKSSLSRVPSFTKGFL
AN4706.1  	----------SAIARSHVAFSDAGSRKGSGAYTTG----DLHRMPSNTKGFL
          	           .  ..:         .  .         .: * ** ****:
```
